# Effectiveness of an Activity Tracker- and Internet-Based Adaptive Walking Program for Adults: A Randomized Controlled Trial

**DOI:** 10.2196/jmir.5295

**Published:** 2016-02-09

**Authors:** Josée Poirier, Wendy L Bennett, Gerald J Jerome, Nina G Shah, Mariana Lazo, Hsin-Chieh Yeh, Jeanne M Clark, Nathan K Cobb

**Affiliations:** ^1^ MeYou Health LLC Boston, MA United States; ^2^ Division of General Internal Medicine The Johns Hopkins University School of Medicine Baltimore, MD United States; ^3^ Welch Center for Prevention, Epidemiology, and Clinical Research The Johns Hopkins University Baltimore, MD United States; ^4^ Department of Kinesiology Towson University Towson, MD United States; ^5^ Division of Pulmonary and Critical Care Medicine Georgetown University Medical Center Washington, DC United States

**Keywords:** physical activity, walking, intervention, adaptive, effectiveness, RCT

## Abstract

**Background:**

The benefits of physical activity are well documented, but scalable programs to promote activity are needed. Interventions that assign tailored and dynamically adjusting goals could effect significant increases in physical activity but have not yet been implemented at scale.

**Objective:**

Our aim was to examine the effectiveness of an open access, Internet-based walking program that assigns daily step goals tailored to each participant.

**Methods:**

A two-arm, pragmatic randomized controlled trial compared the intervention to no treatment. Participants were recruited from a workplace setting and randomized to a no-treatment control (n=133) or to treatment (n=132). Treatment participants received a free wireless activity tracker and enrolled in the walking program, Walkadoo. Assessments were fully automated: activity tracker recorded primary outcomes (steps) without intervention by the participant or investigators. The two arms were compared on change in steps per day from baseline to follow-up (after 6 weeks of treatment) using a two-tailed independent samples *t* test.

**Results:**

Participants (N=265) were 66.0% (175/265) female with an average age of 39.9 years. Over half of the participants (142/265, 53.6%) were sedentary (<5000 steps/day) and 44.9% (119/265) were low to somewhat active (5000-9999 steps/day). The intervention group significantly increased their steps by 970 steps/day over control (*P*<.001), with treatment effects observed in sedentary (*P*=.04) and low-to-somewhat active (*P*=.004) participants alike.

**Conclusions:**

The program is effective in increasing daily steps. Participants benefited from the program regardless of their initial activity level. A tailored, adaptive approach using wireless activity trackers is realistically implementable and scalable.

**Trial Registration:**

Clinicaltrials.gov NCT02229409, https://clinicaltrials.gov/ct2/show/NCT02229409 (Archived by WebCite at http://www.webcitation.org/6eiWCvBYe)

## Introduction

Over a third of US adults are considered sedentary (taking on average fewer than 5000 steps/day) [1], and the average adult takes only 6540 steps/day [2], well short of the 10,000 steps per day target commonly used in public health campaigns. Behavior change interventions using pedometers have been shown to result in moderate increases in physical activity [3,4]. Steps goals are a key predictor of change, with high goals (eg, 10,000 steps/day) associated with the largest increases in physical activity and intervention effect sizes [3,4]. However, even modest increases in activity can yield clinically significant health benefits: an additional 1000 steps/day has been linked to lower body mass index, lower waist-to-hip ratio, and greater insulin sensitivity [5]. Moreover, such goals may require an increase in activity that is hard to reach for certain individuals (eg, those who are sedentary) or difficult to achieve on a daily basis, raising concerns of poor program adherence and high attrition [6]. Smaller, gradual goals may offer a good alternative to high goals for physical activity interventions.

Previous interventions have explored this approach by tailoring goals to a participant’s physical activity level and increasing them by fixed increments (eg, 10% over baseline every 2 weeks or 400 steps/day every week) [3,7,8]. But fixed increments assume a constant, linear trajectory that is seldom observed in health behavior change. For instance, life events (such as sickness or a change in work schedules) and weather may prevent linear progress typically assumed in structured progressive physical activity programs. Fixed goals fail to take into account natural fluctuations in behavior or adjust accordingly. By contrast, goals that dynamically adapt to an individual’s current activity level promptly respond to changes (in either direction) to remain adequately attainable and potentially keep individuals adherent longer while gradually moving them toward higher levels of activity. The effectiveness of adaptive goals was examined in a study that contrasted them to fixed, high goals. In the intervention for overweight adults, system-generated goals were tailored to the participant’s baseline activity level and adjusted over time to reflect changes in activity. Control group participants received a fixed goal of 10,000 steps/day regardless of physical activity level. Adams et al observed that over 6 months incremental, adaptive goals led to larger increases in physical activity than a fixed daily steps goal of 10,000 steps/day [9].

Adaptive interventions can be effective but have not yet been scaled nor tested in a varied population. A large-scale implementation requires the automation of data collection, goal setting, goal messaging, and feedback. The increasingly sophisticated and popular “activity trackers” from manufacturers such as Fitbit, Jawbone, or Fitlinxx enable the implementation of an adaptive goal-setting mechanic. These activity trackers use multi-axial accelerometers to detect walking or running behavior, including tracking steps similarly to mechanical pedometers. Current activity trackers can wirelessly stream data either to a mobile phone or to a local computer, and from there, send the data to other services or programs. Feedback is provided on the activity tracker itself and/or via other programs with which it is paired. Since the activity data is digital, all goal-setting operations (data download, steps goal calculation, and goal messaging) can be automated in real time using a centralized data store and software. A digital, automated implementation could have a wide reach at low cost and potentially, considerable public health impact.

We developed an adaptive walking intervention (Walkadoo) designed to leverage wireless activity trackers and be highly scalable. The intervention is automated, stand-alone, and does not require in-person meetings or staff time to be delivered (apart from the distribution of activity trackers.) The current study examines the effectiveness of an automated adaptive intervention in increasing steps.

## Methods

### Intervention

**Figure 1:**
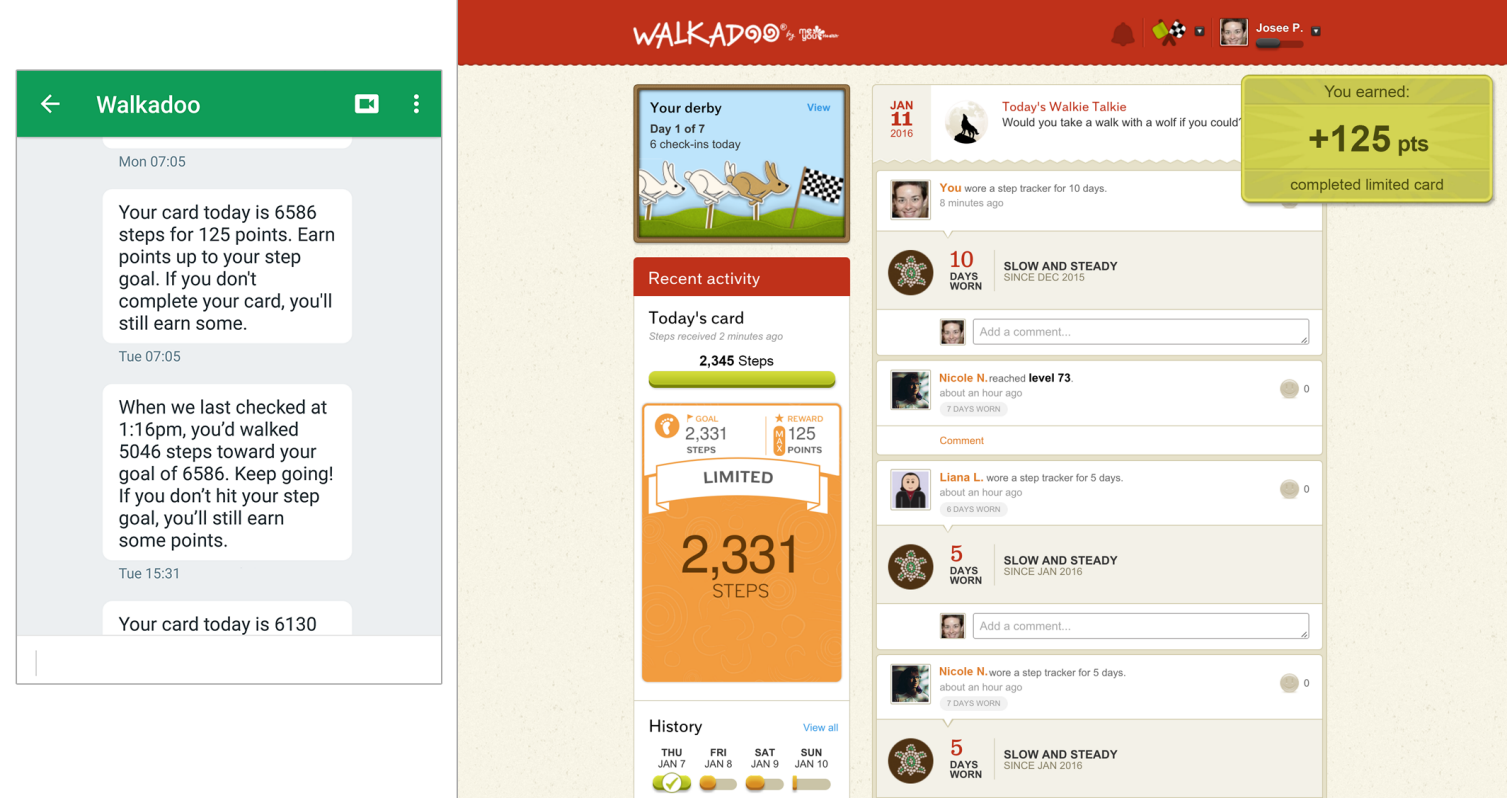
Examples of text messages participants can opt to receive (left) and reward notifications on the main website (right).

Walkadoo [10] is a freely available, open access, Internet-based program that pairs with a range of activity trackers to increase walking behavior. Activity trackers wirelessly and automatically send data to the program throughout the day via sync points, or a Bluetooth connection and the Internet. Participants receive daily steps goals in the morning via email (unless the participant opts out), an optional text message (SMS), on the website, or directly on the activity tracker for those with the option. Participants can opt to receive up to 4 pre-scheduled text messages per day: previous day’s step count, today’s goal, mid-day step count, and/or goal completion notification (see Figure 1). At any time and as often as they like, participants can text the word “steps” to the program to learn the step count after their last data sync and be reminded of their goal for the day, or they can follow their progress through their activity tracker or on the website. Participants receive virtual rewards (points, levels, and badges) for performing certain actions and reaching milestones such as completing a steps goal, achieving a personal best, and engaging socially with the community (eg, by encouraging other participants via “smiles” and comments, or by participating in group competitions) (see Figure 1).

**Figure 2:**
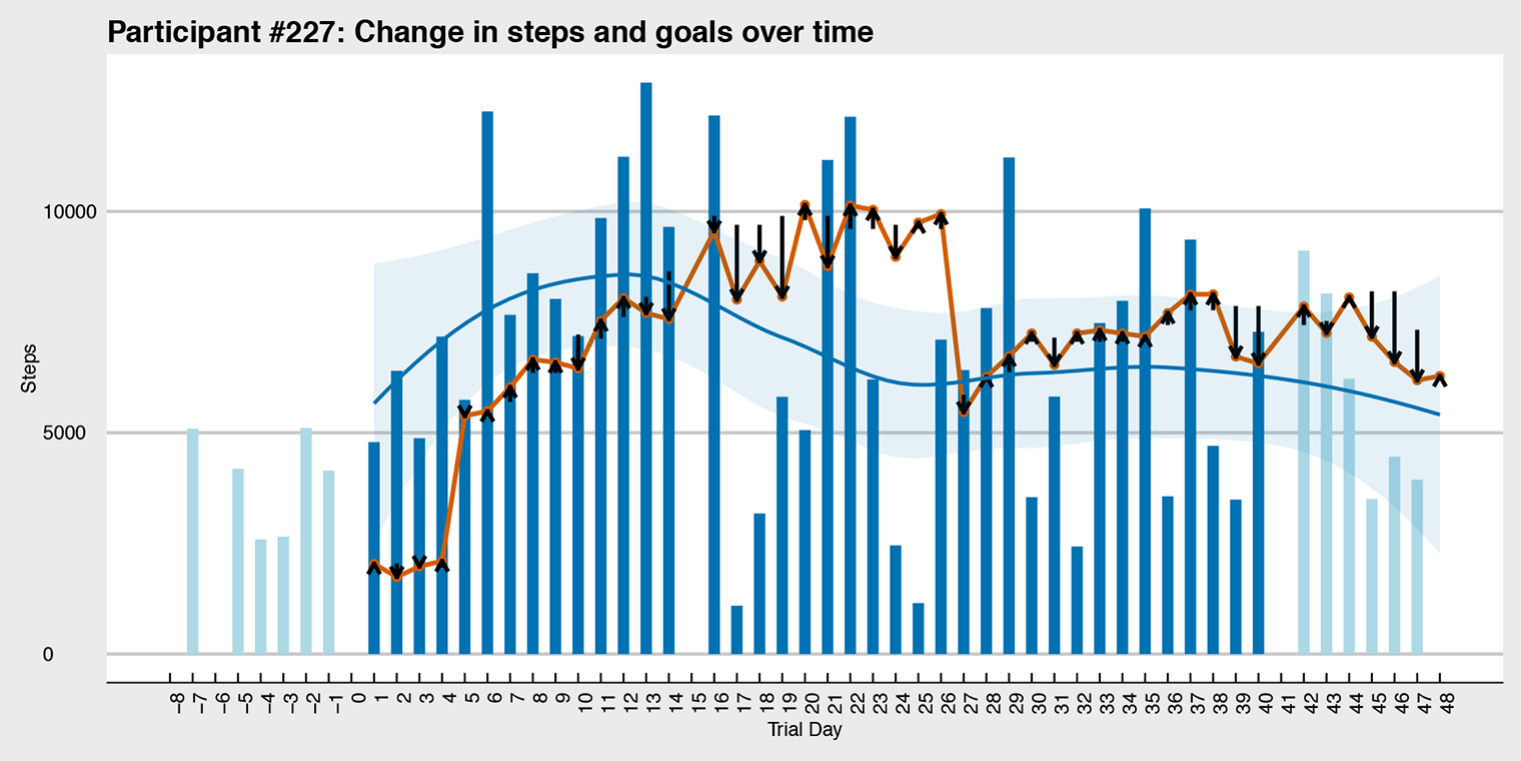
Sample participant steps and goals (actual steps taken are represented by blue bars with an associated trend line and surrounding confidence band; light blue bars indicate run-in data collection and follow-up periods; goals provided to users are represented in red; black arrow markers indicate the direction and magnitude of the random adjustment applied). These random adjustments averaged 2945 steps in either direction. Run-in data are presented here but are not used by the algorithm to preserve generalizability.

The adaptive daily steps goals are the central feature of the program. The system generates goals that are tailored to the participant based on their most recent activity level. The goal-setting algorithm is modeled on a rank-order percentile approach developed following principles of behavioral economics and operant shaping [9]. The approach requires continuous measurements of daily activity to rank, from lowest to highest, the measurements in a 9-day moving window and compute a goal based on a percentile criterion (eg, 60^th^). The program’s algorithm uses a range percentile criterion slightly above the user’s 50^th^percentile, with added algorithmic compensations for insufficient data in the early periods of program use. An additional algorithm randomly selects the exact value of the goal within the range, creating day-to-day variations in difficulty levels that introduce a game-like element of surprise. Figure 2 presents an example of the approach with data from a sample participant during the intervention phase (additional examples can be found in Multimedia Appendix 1.) No major alterations were made to the intervention design over the course of the trial.

### Study Design

The study was a single-site, two-arm randomized controlled trial examining changes in daily steps between a control group asked to continue with their normal routine and an intervention group enrolled in the Walkadoo program. The single-site pragmatic trial was conducted in a real-world workplace setting between early September 2014 and mid-November 2014. It included a 1-week run-in period during which baseline measurements were taken and a 6-week follow-up. The relatively short follow-up aimed to shorten the lag before the learnings from the trial could be fed back into the intervention design cycle in an iterative development model [11]. Schulman Associates Institutional Review Board (IRB) approved the full study protocol and the Johns Hopkins University School of Medicine IRB deemed the subsequent analysis protocol non-human subjects research.

### Recruitment

Participants were employees of Healthways Inc, a multinational company that delivers disease management and well-being improvement solutions. Study recruitment was coupled with standard enrollment in a workplace health program for 599 headquarter-based employees, which included a 3-day program launch onsite event and promotional efforts (such as email announcements and display of posters) that were led by the company’s human resources department. The onsite event marked the start of program availability and the period during which employees could pick up a free activity tracker.

Study recruitment took place during the onsite event. Study staff distributed activity trackers and answered questions that individuals had regarding study participation. Employees interested in participating in the study were instructed to go online to provide informed consent and complete the eligibility check. The study-specific instructions were provided verbally, in a handout, and by email. The beginning and end of study phases were staggered with recruitment over 3 days. Participants incurred no cost to use the activity tracker and/or program.

### Eligibility

A total of 64.8% of employees (388/599) expressed interest and were assessed for eligibility. Individuals logged on to a password-protected website to provide informed consent (participants indicated consent by checking a box and clicking an “I agree to participate” button) and answer a screening questionnaire. Exclusion criteria were failure to complete registration, prior use of Walkadoo, self-reported limited physical mobility, projected lack of Internet access for 4 or more days during the study period, and insufficient activity tracker wear during the run-in period (see below).

### Run-In Period

The run-in period began the day after individuals picked up their activity tracker and lasted 7 days to establish a baseline. Individuals were instructed to wear their activity tracker for at least 12 hours each day. At the end of the 7-day period, participants who met the minimal activity tracker wear criterion (at least 10 hours a day on a minimum of 4 days including 1 weekend day) were randomized.

### Randomization

We generated randomization assignment sequences that were stratified by baseline physical activity levels. The physical activity strata were sedentary (<5000 steps/day on average), low to somewhat active (5000-9999 steps/day on average), and active to highly active (≥10,000 steps/day on average) [12]. The enrollment system randomly allocated participants to either arm in a 1:1 ratio. Participants were notified of their randomization assignment via email and received instructions based on their trial arm and were therefore not blinded.

### Control Group

After the run-in period, participants in the control group were instructed not to wear their activity tracker and to maintain their daily activity routine for 6 weeks (until follow-up.)

### Intervention Group

Intervention participants were provided user accounts and prompted to complete formal registration into Walkadoo*.* Participants were instructed to install the provided universal serial bus (USB) dongle and synchronization software on their home computer, allowing the activity tracker to sync data with the program whether the participant was at work or at home. The visual feedback on the activity tracker (see Measurements for detail) was activated for intervention participants for the remainder of the study.

### Follow-Up

After 6 weeks, all participants received an email asking them to wear their activity tracker for at least 10 hours a day for the next 7 days. Participants without sufficient data (who did not provide data for at least 10 hours a day on a minimum of 4 days including 1 weekend day, the same wear time criterion as for baseline measurements) were granted another 7-day window for a second attempt. All study participants were allowed to keep the activity tracker at the end of the study, while only participants who completed follow-up received a US $25 Amazon gift card as compensation for their time.

### Measurements

Primary outcome measures were steps recorded by the activity tracker. Steps were estimated using the Pebble+ (Fitlinxx Inc), a commercially available wireless accelerometer designed to be worn on the hip or shoe. An earlier version of this activity tracker had been shown to have similar accuracy to research-grade accelerometers (YAMAX and Actigraph) during treadmill and over ground walking (from 2-8 mph) [13]. Step data automatically offloaded throughout the day via wireless sync points that were positioned on each floor so as to cover the whole office area. Visual feedback on the activity tracker (a circle that gradually lit up to indicate relative progress toward the day’s steps goal, without a step count) was disabled at baseline for both study arms and was enabled for the rest of the study in the intervention arm only. The activity tracker reports data in 20-minute increments. Wear time was estimated from the earliest and latest moments of activity during the day. Process data including site visits and email opens were collected for the intervention arm.

### Analysis

The primary outcome was the difference between arms in the change in steps per day from baseline to follow-up. Mean steps per day were calculated as the total number of steps taken on valid days (ie, with at least 10 hours of wear time) divided by the number of valid days (range 4-7 days). A subgroup analysis was planned to examine change in steps per baseline activity level as stratified. The secondary outcome was the difference between arms in the proportion of study participants who increased their steps per day by 1000 steps, which is the smallest change in activity that has been linked to health outcomes [5].

Statistical significance for the between-group difference in change in steps per day from baseline to follow-up (primary outcome) was assessed using a two-tailed independent-samples *t* test. A priori we decided to report the *t* test as the main analysis to estimate the mean differences in change in steps per day by study arm. To evaluate the robustness of the unadjusted analysis, we used a repeated-measures mixed-effect model with all available baseline and follow-up data that met the activity tracker wear time requirement. We estimated the mean difference from baseline to follow-up as a function of group assignment and adjusted for age, race, and gender and baseline physical activity stratum. The proportional difference for increases of 1000 steps/day between study arms (secondary outcome) was assessed using a chi-square test. We conducted a sensitivity analysis to evaluate for significant selection bias by participants lost to follow-up. We re-calculated the main analysis using all available follow-up data over the 2 assessment weeks, eliminating the minimum follow-up data requirement. Analyses were performed using SAS, version 9.4. Significance level was set at *P*<.05 for all analyses.

## Results

### Participant Characteristics

**Figure 3:**
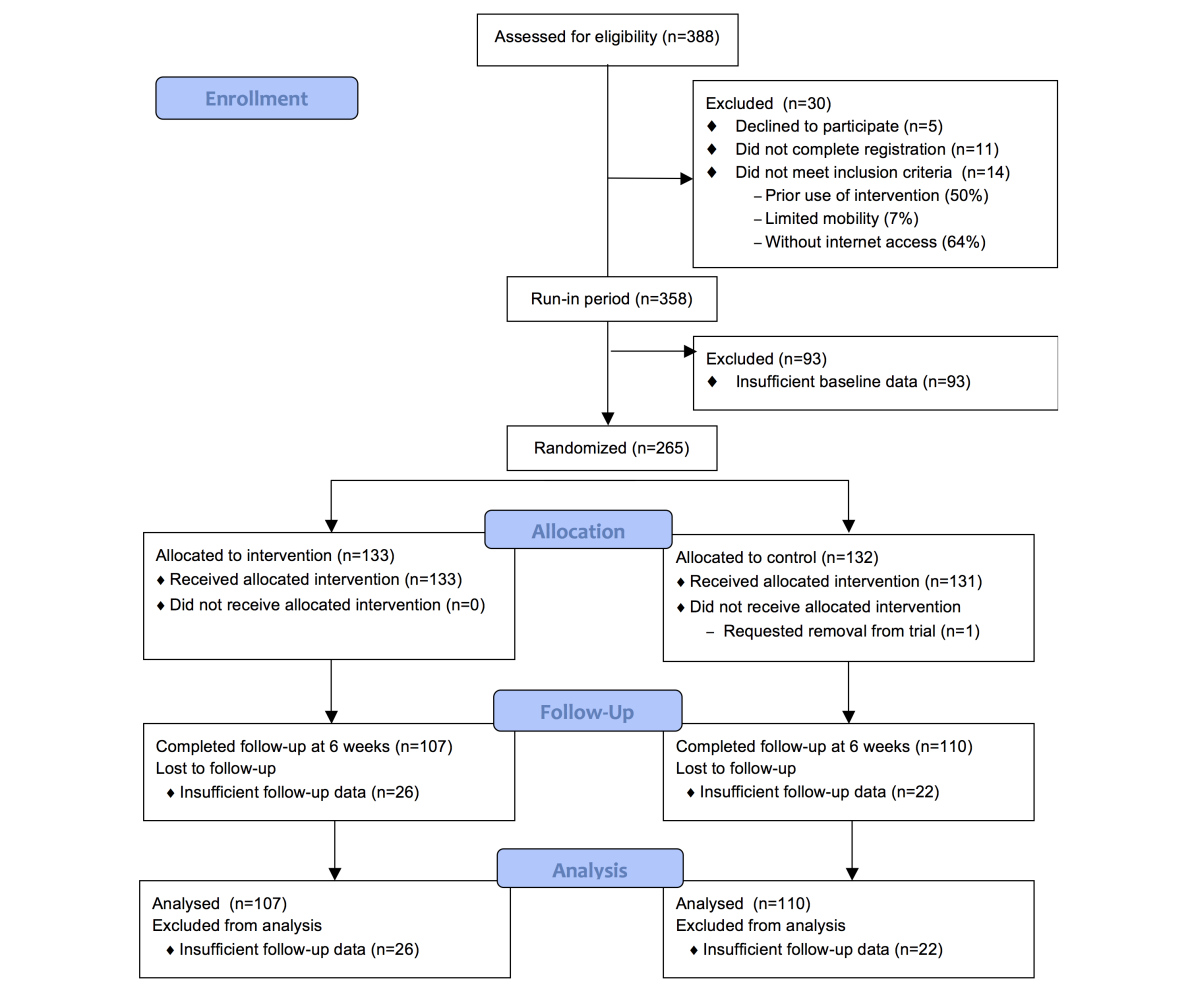
CONSORT diagram.

A total of 388 employees were assessed for eligibility with 30 excluded due to no informed consent, incomplete registration, or not meeting inclusion criteria. Among the 358 candidates who completed the run-in period, 93 were excluded because they failed to meet the minimum activity tracker wear criterion. There were 265 participants randomized to the Walkadoo intervention (n=133) and the control arm (n=132) (see Figure 3).

The baseline characteristics of the study population overall and by arm are shown in Table 1.

Overall, two-thirds (175/265, 66.0%) were women and one-third (90/265, 31.3%) had an annual household income of less than US $60,000. Physical activity level was classified as sedentary for over half (142/265, 53.6%) of participants and low to somewhat active for (119/265, 44.9%). During baseline data collection, participants wore their activity tracker for at least 10 hours on an average of 6.4 days, with an overall average of 14.4 hours/day (see Table 1). The two arms did not differ in their wear time at baseline (control: mean 14.6, SD 1.3; intervention: mean 14.4, SD 1.1, *P*=.23) or at follow-up (control: mean 14.4, SD 1.3; intervention: mean 14.7, SD 1.6, *P*=.21).

We collected complete follow-up data for 217 (81.9%, 217/265) participants. The 48 participants without complete data were similar to those with complete data in terms of baseline physical activity level, race/ethnicity, income, and education (see Multimedia Appendix 2).

Indicators of program participation in the treatment group are presented in Table 2. Participants wore their activity tracker on 78.6% of days (33.0/42 days) on average. Participants opened 21.9% of their daily emails (9.2/42 days) and visited the website every 3.6 days on average (11.8/42 days). The opening of text messages cannot be tracked and is not reported. Participants completed their steps goals on average on 18.3 days (SD 6.6, IQR=7) out of 42. In the sixth and last week of treatment, 97.7% (130/133) of intervention participants still wore their activity tracker, opened emails, and/or visited the website.

### Effect of the Intervention

From baseline to follow-up, participants in the intervention arm increased their activity by a mean of 309 steps/day (SD 1874). Activity in the control arm decreased by a mean of -661 steps/day (SD 1824). Change over baseline statistically differed between the intervention and control arms (difference=970 steps/day; *P*<.001; see Table 3). The repeated-measures model confirmed a statistically significant difference in change from baseline between the two arms, with the intervention group showing an increase of 845 steps/day over control (arm x time point interaction, *P*<.001, 95% CI 463-1228).

**Table 1:** Baseline characteristics of randomized participants.

		Total (N=265)	Control (n=132)	Intervention (n=133)	*P* value^a^
Age in years, mean (SD)		39.9 (11.7)	39.6 (12.0)	40.3 (11.4)	.65
Women, n (%)		175 (66.0)	92 (69.7)	83 (62.4)	.21
**Race/ethnicity, n (%)**					.99
	White	205 (77.4)	101 (76.5)	104 (78.2)	
	Black	30 (11.3)	15 (11.4)	15 (11.3)	
	Hispanic	4 (1.5)	2 (1.5)	2 (1.5)	
	Asian	11 (4.2)	5 (3.8)	6 (4.5)	
	Other	7 (2.6)	4 (3.0)	3 (2.3)	
	Don’t know	8 (3.0)	5 (3.8)	3 (2.3)	
**Education, n (%)**					.81
	High school or vocational school	11 (4.1)	7 (5.3)	4 (3.0)	
	Some college	30 (11.3)	14 (10.6)	16 (12.0)	
	College graduate	124 (46.8)	61 (46.2)	63 (47.4)	
	Post-graduate	98 (37.0)	49 (37.1)	49 (36.8)	
	Don’t know/Prefer not to answer	2 (0.8)	1 (0.8)	1 (0.8)	
**Annual income, $US**					.61
	<$60,000	83 (31.3)	45 (34.1)	38 (28.6)	
	$60,000-$120,000	73 (27.6)	34 (25.8)	39 (29.3)	
	> $120,000	56 (21.1)	27 (20.4)	29 (21.8)	
	Don’t know/Prefer not to answer	53 (20.0)	26 (19.7)	27 (20.3)	
**Baseline physical activity level, n (%)**					.99
	Sedentary (<5000 steps/day)	142 (53.6)	71 (53.8)	71 (53.4)	
	Low to somewhat active (5000-9999 steps/day)	119 (44.9)	59 (44.7)	60 (45.1)	
	Active to highly active (≥10,000 steps/day)	4 (1.5)	2 (1.5)	2 (1.5)	
Number of valid^b^ days, mean (SD)		6.4 (0.8)	6.3 (0.8)	6.4 (0.8)	.51
Hours of wear per day, mean (SD)		14.4 (1.2)	14.4 (1.3)	14.4 (1.1)	.68
Has 2 (vs 1) valid^b^ weekend days, n (%)		172 (64.9)	88 (66.7)	84 (63.2)	.55

^a^Comparisons were performed by chi-square tests for categorical variables and independent samples two-tailed *t* tests (means) and Wilcoxon rank sum tests (medians) for continuous variables.

^b^A valid day is defined as having at least 10 hours of activity tracker wear time.

**Table 2:** Indicators of program use for participants in the intervention arm (n=133): number of days (of 42) that participants wore their activity tracker (as shown by >100 steps recorded), opened their daily email at least once, and visited the website at least once.

	Activity tracker worn	Email opened	Website visited
Mean (SD)	33.0 (11.6)	9.2 (10.4)	11.8 (11.2)
Range	0-42	0-42	0-39
IQR	12	14	19

We conducted the pre-specified stratified analyses by baseline activity level stratum (see Table 3). Among the sedentary group, the intervention arm had a mean increase of 595 steps/day (SD 1558), which was statistically significantly higher than the control arm (47 steps/day, SD 1299, *P*=.04). The low to somewhat active group decreased regardless of treatment but significantly more so in the control arm (intervention: -110 steps/day; control: -1286 steps/day, *P*<.001).

In a sensitivity analysis, we evaluated primary outcome on a sample including an additional 35 participants who had some available follow-up data but had failed to meet the minimal activity tracker wear criterion. In this sample of participants (252/265, 95.1%), the 130 participants in the control arm reduced their mean steps per day by -753 (SD 1836) while the 122 participants in the intervention arm increased their mean steps per day by 80 (SD 1999). The statistically significant between-group difference (*P*<.001) was consistent with the primary findings.

Finally, participants in the intervention arm were more likely to achieve an increase of 1000 steps/day as compared with the control arm (n=32 or 29.9% vs n=18 or 16.4%, respectively, *P*=.018).

**Table 3:** Steps/day at baseline and follow-up, and change from baseline to follow-up among participants who met the minimum activity tracker wear criterion^a^ for follow-up data collection (n=217).

Physical activity at baseline^b^		Control, mean (SD)	Intervention, mean (SD)	*P* value^c^
**All (control n=110; intervention n=107)**				
	Baseline	5412 (2251)	5102 (1901)	.27
	Follow-up	4751 (1834)	5411 (2277)	.02
	Change from baseline to follow-up	-661 (1824)	309 (1874)	<.001
**Sedentary (<5000 steps/day) (control n=59; intervention n=58)**				
	Baseline	3820 (1061)	3769 (970)	.79
	Follow-up	3867 (1654)	4363 (1517)	.09
	Change from baseline to follow-up	47 (1299)	594 (1558)	.04
**Low to somewhat active (≥5000-9999 steps/day) (control n=49; intervention n=48)**				
	Baseline	6992 (1275)	6580 (1310)	.12
	Follow-up	5706 (1466)	6470 (2075)	.04
	Change from baseline to follow-up	-1286 (1783)	-110 (2106)	.004

^a^Minimum activity tracker wear criterion for follow-up data collection required 4 days with at least 10 hours of activity tracker wear time including 1 weekend day.

^b^Per-stratum comparisons excluded the 3 participants who had 10,000+ steps/day at baseline.

^c^Comparisons performed with independent samples two-tailed *t* tests.

## Discussion

### Principal Findings

We evaluated an intervention designed to increase steps using daily adaptive goals tailored to an individual’s current activity level. In a worksite environment, the walking program increased steps by a mean difference of 970 steps/day over control. This magnitude, while modest, has been previously correlated with improvements in body mass index and insulin sensitivity over time [5].

The findings were observed in both sedentary (<5000 steps/day) and non-sedentary (5000-9999 steps/day) individuals. Sedentary individuals represent 36.1% of the US population and are more likely to have multiple risk factors such as smoking or obesity [1], making them a critical population for public health programs. Of note, only 4 participants (1.5% total; two in each arm) were classified as active to highly active at baseline (taking at least 10,000 steps/day), as compared to 16.3% of Americans in the 2005-2006 NHANES cohort [1]. Active to highly active individuals may not have been interested in a walking program or may have been discouraged from participating in the trial if they had another activity tracker, since participants were asked to refrain from using activity trackers other than the one provided for the trial. Our results suggest that an adaptive walking program has the potential to benefit broad segments of the population as 83.7% of US adults take <10,000 steps/day [1].

The between-group difference at follow-up was partially driven by a decrease in steps in the control group. Baseline activity might have been higher due to reactivity (an immediate and temporary increase in physical activity due to wearing an activity tracker.) A reactivity effect has been previously reported, although it is unusual with “sealed” activity trackers with inactive or hidden visual feedback [14]. The decline in steps from baseline to follow-up we observed may represent a regression to a true baseline behavior as reactivity wore off. Alternatively, the intervention may have attenuated the known seasonal decline in light physical activity between summer and fall [15], when the trial was conducted. Like previous investigators, we have no way to verify either hypothesis conclusively, although the findings reinforce the importance of randomized controlled designs for testing the effectiveness of behavior change interventions.

The program showed convincing engagement levels. Participants wore their activity tracker on most (78.6%) days and remained active into their sixth week of treatment (77.7% interacted with the program at least once). Email open rates were tracked by the use of an embedded image that may be suppressed by certain email clients and underestimate actual rates. It is worth noting that participants could receive their daily steps goals in several ways other than opening the email: the steps goal could be read in the email subject line itself, received and requested by text message, found on the website, or tracked on the activity tracker. However, there are no standard metrics available for direct comparison. We encourage researchers to report intervention usage data so reference points can be found in the literature.

A strength of this trial was its pragmatic approach in a real-world workplace setting. We recruited trial participants from an employee population who received Walkadoo as part of their workplace wellness program offering. Our findings add to the evidence that physical activity interventions can be effective in the workplace [16-18] where employees tend to sit at their desks for long periods. This program was effective despite not being designed specifically or exclusively for workplace implementations. We demonstrated that an adaptive program can be automated and made scalable using a simple wireless activity tracker.

Despite the pragmatic approach, several limitations to this trial should be noted. The chosen study population was one of convenience and the generalization of our findings will require extension and replication in future work. With respect to measurements, the manufacturer’s directions for wearing the activity tracker indicated it could be worn on the hip or on the shoe. Although placement may limit the comparison of steps with more standardized methods and devices, our analyses appropriately focused on individual change scores. As part of the pragmatic approach we used a relatively short follow-up period to ensure prompt availability of the results to program development teams, evaluators, and purchasers [11]. Still, sustainability of the effect remains to be demonstrated.

### Conclusions

The evolution of mechanical pedometers to digital activity trackers has opened the doors for interventions, such as Walkadoo*,* that leverage real-time­­ access to data, predictive analytics, and algorithmic detection of activity patterns. While widely available activity trackers promote exercise monitoring, their largest public health impact could be on simple walking activity.

The results of this pragmatic trial confirm that dynamic programs tailored to the individual are a realistic and scalable alternative to fixed goals and that they can be effective in shifting health behavior in a real-world population. Future interventions will also be able to draw from the rich dataset provided by modern activity trackers to set goals that are not just tailored to the individual, but also adapt in real time to behavior or the environment, such as weather or physical geo-location. Perhaps more importantly, newer activity trackers, including the most recent generation of mobile phones, can detect more complex activities, such as stair climbing, while also providing the resolution to detect periods of sedentary behaviors (sitting or inactivity). Programs that can effect change across such an array of active and inactive behaviors could directly impact public health for adults who move too little or sit too much. Such an elusive but promising potential merits additional research and attention.

## Appendix

Multimedia Appendix 1 Examples from participants.

Multimedia Appendix 2 Supplemental table.
