# Supplementary material for: Effectiveness of an Activity Tracker- and Internet-Based Adaptive Walking Program for Adults: A Randomized Controlled Trial
Source: J Med Internet Res. 2016 Feb 9;18(2):e34. doi: 10.2196/jmir.5295 (PMC4764789; doi:10.2196/jmir.5295)

Multimedia Appendix 1.

Participant #86 shows a significant ramp-up in steps early in the intervention period that is essentially sustained through follow-up. The first 4 days provide default values (2,000 steps) that the participant does not meet, causing goals to drop on day 5 just as their steps begin to increase. The goals catch up with behavior on day 11.

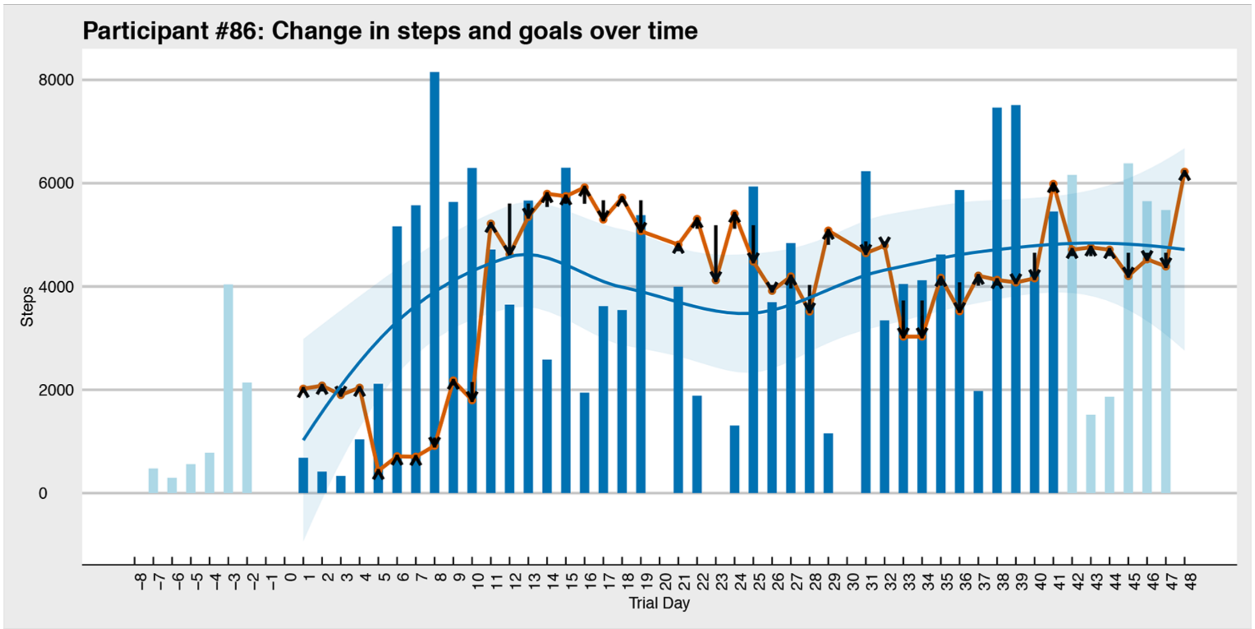

Participant #155 shows an initial increase in steps followed by a slow decline over the rest of the trial. Notably, default goals at the start of the intervention phase are lower than the user's baseline from the run-in period.

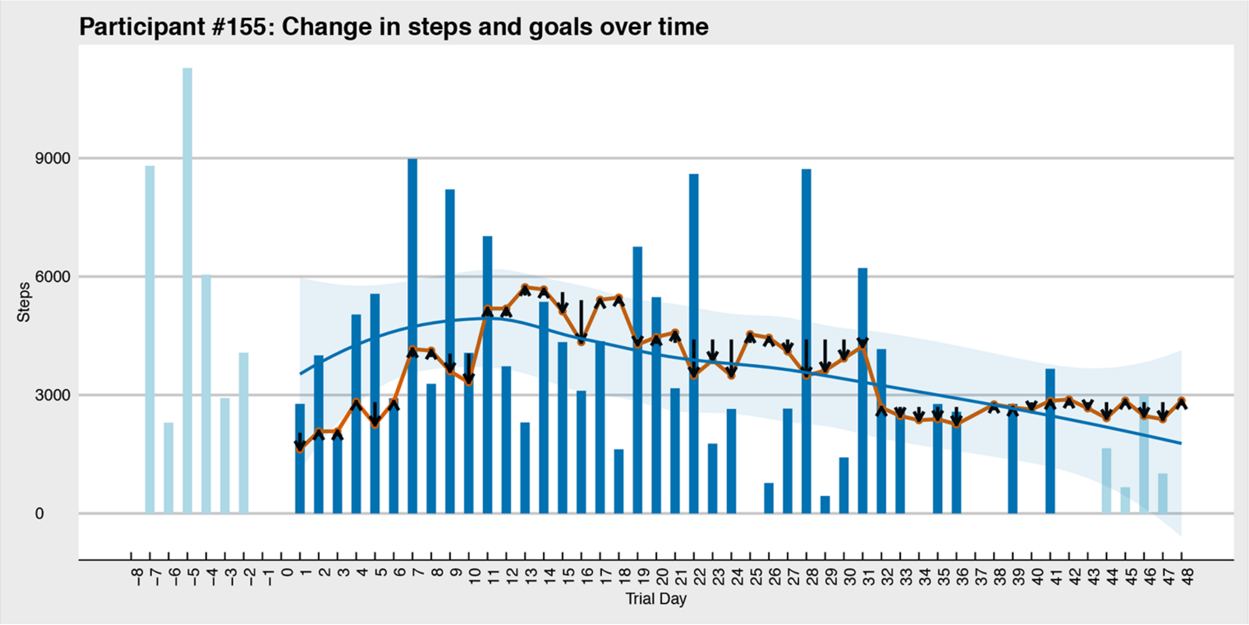

Participant #336 shows an initial increase in steps and matching goal adjustment, followed by a decrease in steps and secondary adjustment. By the end of the intervention phase their goals have stabilized.

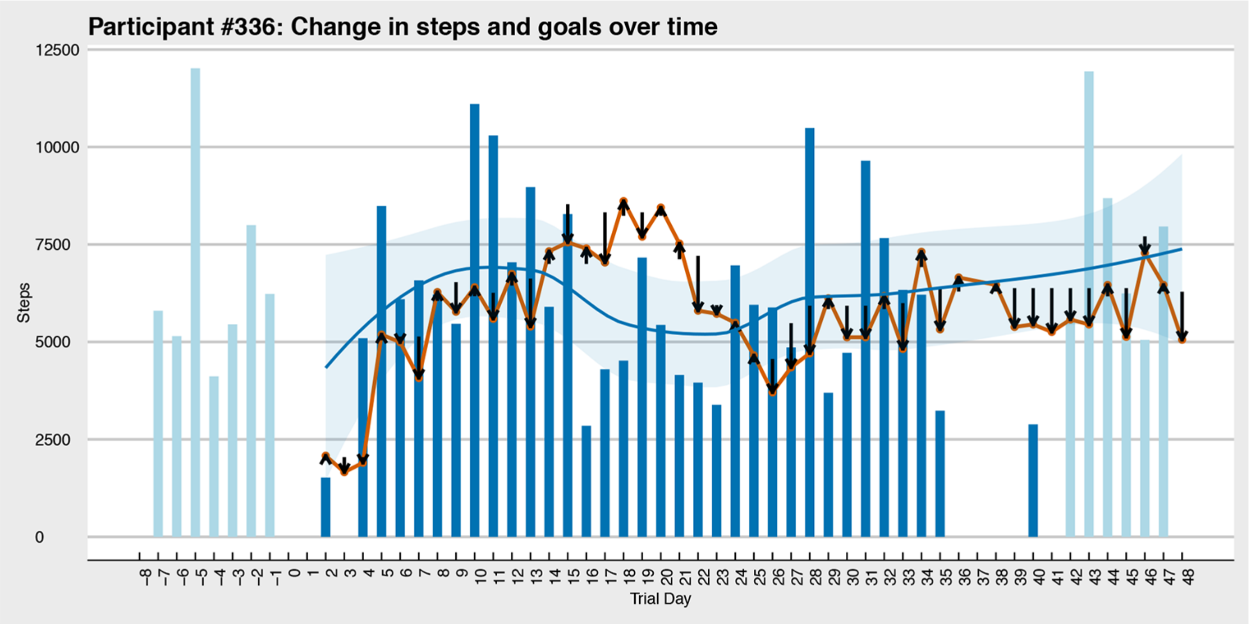

Participant #145 shows a gradual increase in steps and goals over the first 21 days of the trial with a number of days with missing data (where they did not wear their activity tracker). They become fully non-adherent on day 28 but are not lost to follow-up as they wear their activity tracker for the follow-up phase.

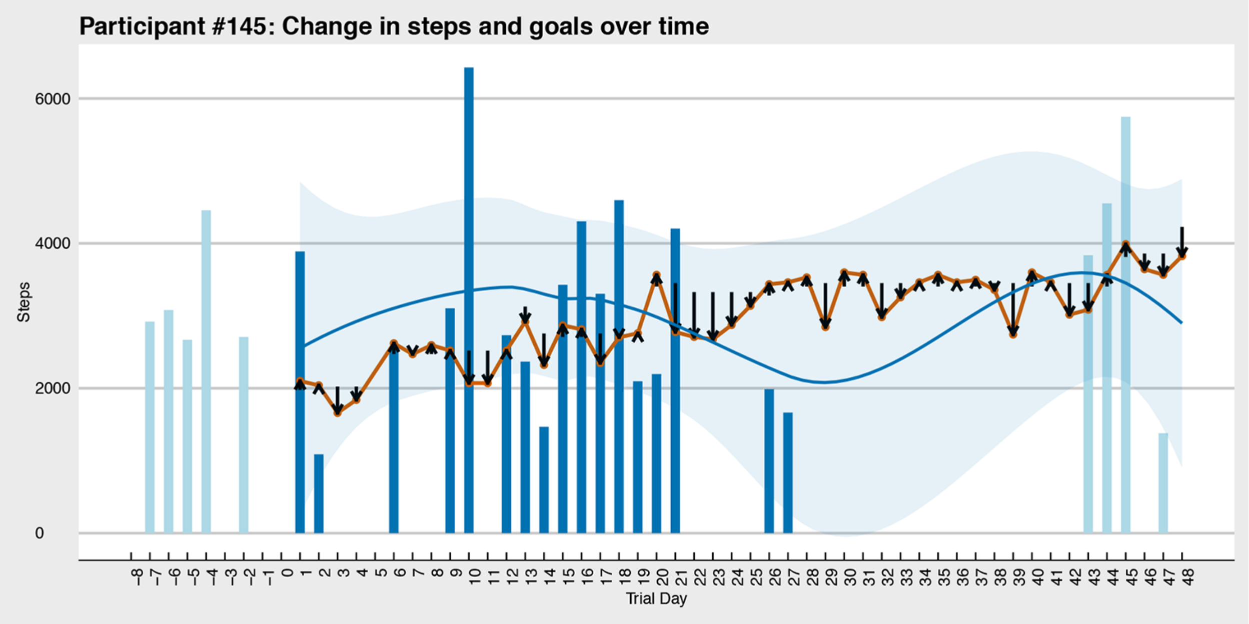

Participant #52 demonstrates the goal algorithms's dependence on daily data synchronization from the activity tracker. While this participant wore their activity tracker consistently through the trial they did not synchonize their data on days 17-25 (not indicated in the figure). Since the algorithm uses the trailing 9 days of valid data the algorithm was unable to adjust to the user's new pattern until they synchronized and uploaded those trailing 8 days of information on day 26. This causes an abrupt drop in goals for day 27 as the algorithm takes into account the newly available data.

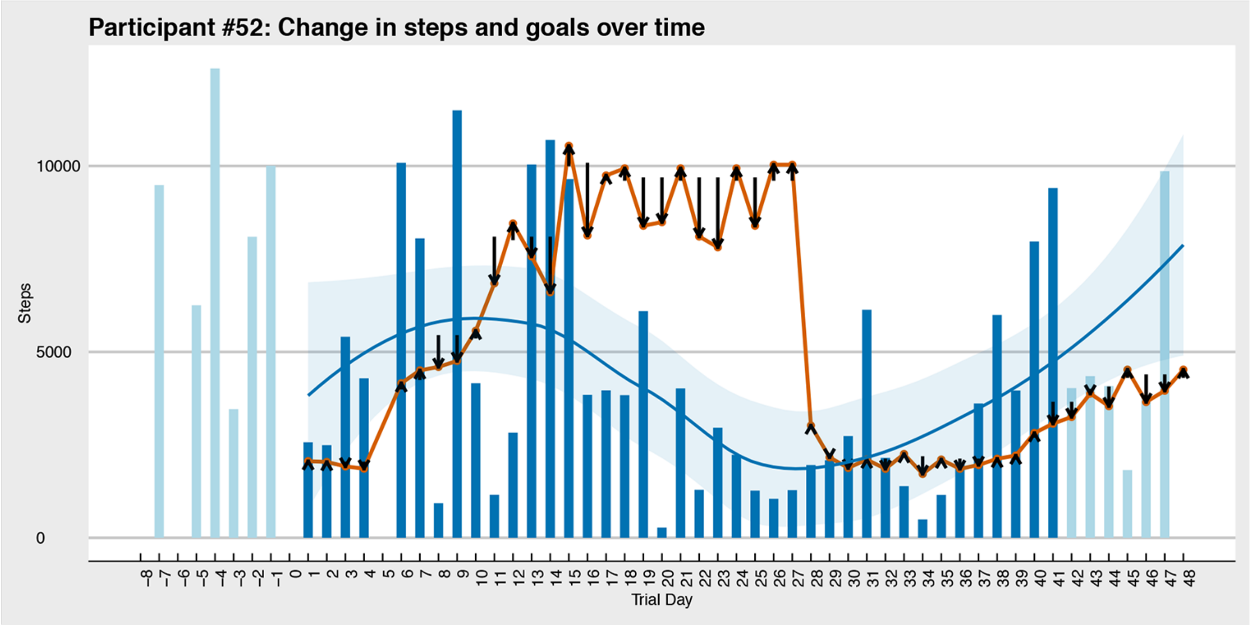

Supplement: Multimedia Appendix 1 [file jmir_v18i2e34_app1.pdf]
