# Supplementary material for: Effectiveness of an Activity Tracker- and Internet-Based Adaptive Walking Program for Adults: A Randomized Controlled Trial
Source: J Med Internet Res. 2016 Feb 9;18(2):e34. doi: 10.2196/jmir.5295 (PMC4764789; doi:10.2196/jmir.5295)
Supplement: Multimedia Appendix 2 [file jmir_v18i2e34_app2.pdf]

Multimedia Appendix 2. Comparison of baseline characteristics between participants with complete (n=217) and incomplete follow-up data (n=48).

	**Non-Completers n=48**	**Completers n=217**	**p-value[1]**
Study arm			0.54
Intervention	26 (54.2)	107 (49.3)	
Control	22 (45.8)	110 (50.7)	
Measured physical activity at baseline			0.76
Sedentary (<5,000 steps/day)	25 (52.1)	117 (53.9)	
Low to somewhat active (5,000-9,999 steps/day)	22 (45.8)	97 (44.7)	
Active to highly active (10,000+ steps/day)	1 (2.1)	3 (1.4)	
Activity tracker wear at baseline			
Total valid[2] days at baseline (4-7 days)	6.0 ± 1.0	6.5 ± 0.7	0.003
Wear time per day, hours			
Mean ± SD	14.5 ± 1.6	14.4 ± 1.6	0.83
Median (1st, 3rd IQR)	14.5 (13.2, 15.8)	14.3 (13.0, 15.3)	0.76
Age in years			
Mean ± SD	36.7 ± 12.1	40.6 ± 11.5	0.035
Median (1st, 3rd IQR)	33 (28, 43)	39 (31, 50)	0.024
Gender			0.92
Male	32 (66.7)	143 (65.9)	
Female	16 (33.3)	74 (34.1)	
Race			0.71
White	36 (75.0)	169 (77.9)	
Black	6 (12.5)	24 (11.1)	
Hispanic	0	4 (1.8)	
Asian	3 (6.2)	8 (3.7)	
Other	2 (4.2)	5 (2.3)	
Don’t know	1 (2.1)	7 (3.2)	
Education level			0.07
High School, Technical/vocational school	4 (8.3)	7 (3.2)	
Some college	3 (6.3)	27 (12.4)	
College graduate	28 (58.3)	96 (44.2)	
Post graduate	13 (27.1)	85 (39.2)	
Don’t know/Prefer not to answer	0	2 (1.0)	
Annual income			0.45
< $60,000	20 (41.7)	63 (29.0)	
$60,000 - $120,000	12 (25.0)	61 (28.1)	
> $120,000	10 (20.8)	46 (21.2)	
Don’t know/Prefer not to answer	6 (12.5)	47 (21.7)	
Number of valid[2] days, mean ± SD	6.0 ± 1.0	6.5 ± 0.7	0.003
Hours of wear per day, mean ± SD	14.0±1.4	14.5 ± 1.2	0.015
Has 2 (vs 1) valid[2] weekend days, n (%)	23 (47.9)	149 (68.7)	0.006

1. Comparisons were performed by $\chi 2$ tests for categorical variables, and independent samples two-tailed t-tests (means) and Wilcoxon rank sum tests (medians) for continuous variables.
2. A valid day is defined as having at least 10 hours of activity tracker wear time.
